# Donor neo-atrial cuff construction after accidental lower lobe vein transection

**DOI:** 10.1186/s13019-022-02013-3

**Published:** 2022-10-04

**Authors:** Raphael S. Werner, Claudio Caviezel, Isabelle Opitz, Ilhan Inci

**Affiliations:** Department of Thoracic Surgery, University Hospital Zurich, Rämistrasse 100, 8091 Zurich, Switzerland

**Keywords:** Lung transplantation, Donor lung, Reconstruction, Left atrial cuff, Pulmonary vein

## Abstract

**Background:**

An inadequate donor left atrial cuff is a rare technical issue after graft procurement for lung transplantation. With regard to the shortage of suitable donor organs for lung transplantation, these organs should be surgically reconstructed to avoid the loss of an organ and a futile intervention in the critically ill recipient.

**Case presentation:**

We report a case of a 62-year old patient who underwent bilateral sequential lung transplantation for chronic obstructive pulmonary disease. During isolated lung procurement, the right inferior pulmonary vein was circumferentially transsected and separated from the right superior pulmonary and middle lobe veins. Subsequently, a reconstruction of the left atrial cuff with an acellular biological patch was performed to complete the atrium anastomosis. The patient experienced an uneventful postoperative recovery and a follow-up ventilation/perfusion scan showed normal perfusion of the right lower lobe.

**Conclusions:**

This case demonstrates that reconstruction of an inadequate left atrial cuff with a biological patch is feasible and allows for an adequate venous drainage and therefore normal transplant organ function.

## Background

In adequately selected patients with end-stage chronic pulmonary disease, lung transplantation offers a valuable treatment option [1]. A standard procedure involves a single anastomosis between the donor left atrial cuff and the clamped recipient left atrium. On rare occasions surgeons may encounter an inadequate donor left atrial cuff for a safe anastomosis, despite proximal placement of the Satinsky clamp on the recipient left atrium. This may either be due to anatomical abnormalities of the donor pulmonary vasculature, or due to technical problems during procurement or back-table preparation [2]. Especially when the heart is harvested beforehand, the pulmonary venous confluence may be left with only little or no surrounding atrial tissue [3]. Cases with a severely deficient left atrial cuff may even enforce a discontinuation of the transplant procedure. The implanting surgeons may therefore be confronted with the situation to repair the left atrial cuff in order to continue the transplantation.

Here we report the case and technique of a bilateral lung transplantation with inadequate donor left atrial cuff due to an accidentally transected lower lobe vein. This part is reconstructed with an acellular biological patch (bovine pericardium). For the review of clinical records and the publication of this case, an informed consent was obtained from the patient.

## Case presentation

**Fig. 1:**
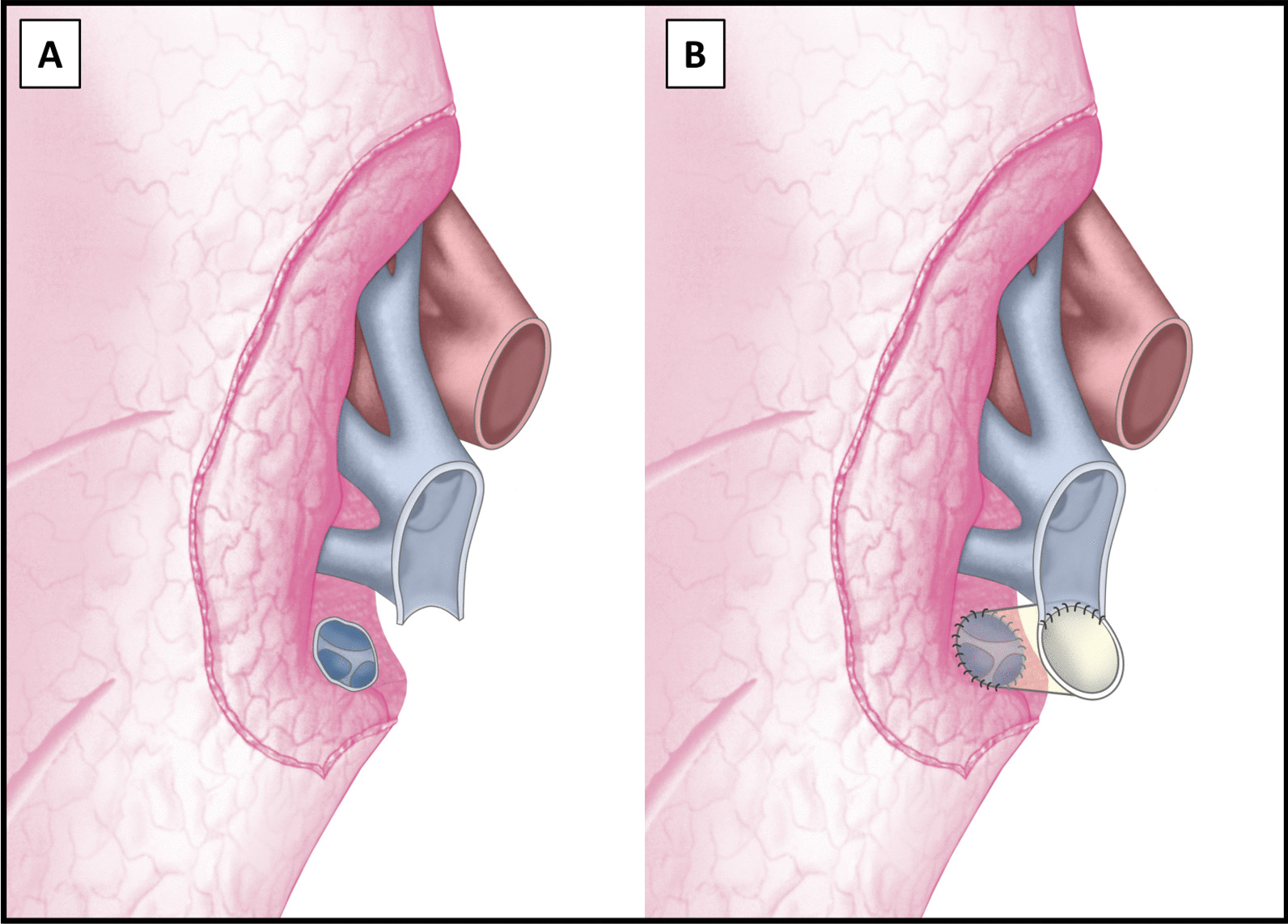
Schematic presentation of the retracted cuff around the right lower pulmonary vein after lung procurement (**A**) and the reconstructed neo-atrial cuff (**B**)

A 62 year old male patient with chronic obstructive pulmonary disease GOLD grade 4, centrilobular lung emphysema, normal alpha-1 antitrypsin levels and oxygen therapy during exertion was listed for bilateral lung transplantation. He had a smoking history of 80 pack years, but had stopped smoking 6 years before listing. A video-assisted lung volume reduction surgery 4 years prior to listing had resulted in a significant improvement in the patient’s exercise capacity and forced expiratory volume in one second (FEV1). The beneficial effect lasted for 2 years, until a further decrease in his pulmonary function occurred. Before listing, forced vital capacity (FVC) was 1.45L (38% predicted), FEV1 was 0.47L (16% predicted) and FEV1/FVC was 32%. An examination by right heart catheter revealed a mild precapillary pulmonary hypertension with a mean pulmonary artery pressure of 34 mmHg. One month after listing, a suitable donor organ from a 75-year old non-smoker was allocated. A chest computed tomography (CT) of the donor showed normal parenchymal structure and bronchoscopy revealed a clear bronchial tree. The graft procurement was performed according to a standard procedure [4]. No heart procurement was performed. During lung procurement, the atrial cuff around the orifice of the right inferior pulmonary vein was inadvertently cut too short leading to a retraction of the remaining vein into the pulmonary hilum (Fig. 1A).

**Fig. 2:**
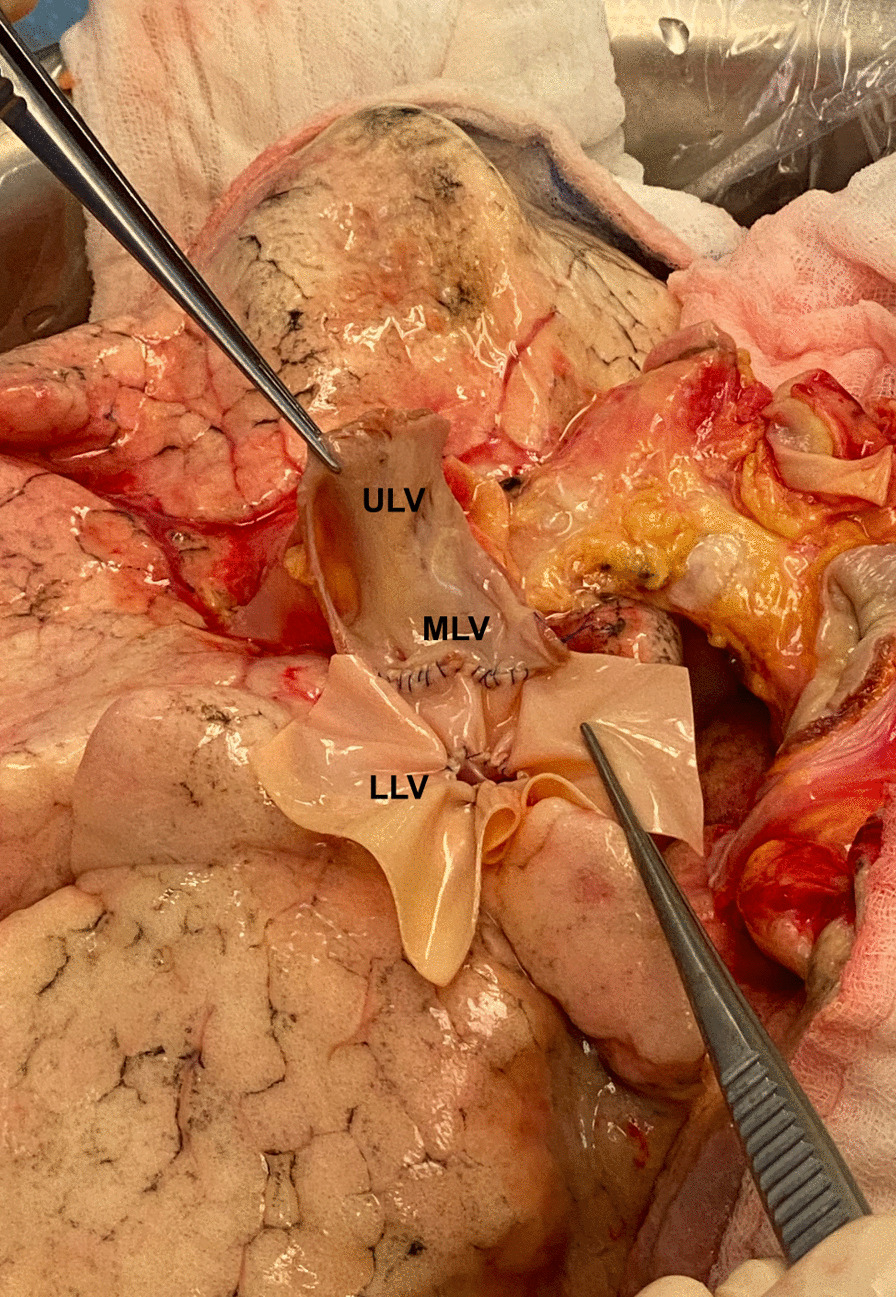
The reconstructed atrial cuff of the right lower lobe vein (LLV), sutured to the cuff of the upper lobe vein (ULV). *MLV* middle lobe vein

**Fig. 3:**
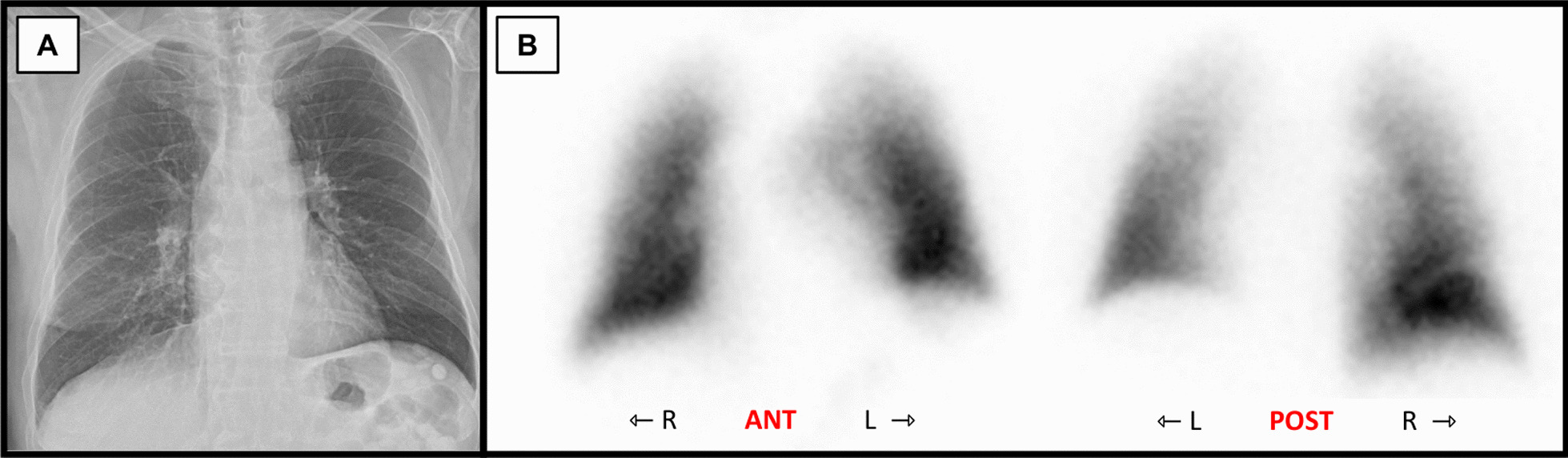
**A** Postoperative x-ray without signs of pulmonary congestion. **B** A V/Q scan on the 29th postoperative day showed symmetrical perfusion of the donor lung

Upon arrival at the implant site, the lung block was carefully inspected on the back table and the pulmonary veins were flushed with preservation fluid in a retrograde fashion. The right inferior pulmonary vein was circumferentially amputated, separated from the right superior pulmonary vein and retracted into the hilum. Since the inadequate size of the donor left atrial cuff was not noticed during procurement, no additional pericardial or atrial donor tissue was brought along for the reconstruction of the atrial cuff. While a reconstruction with allogeneic pericardial tissue would have been preferred, our reconstruction was therefore performed using an acellular, biological patch (Supple Peri-Guard^®^, Lamed, Germany). The patch was cut out centrally to match the pulmonary vein’s orifice and sutured to the remaining cuff using a 4–0 polypropylene running suture. The biological patch was then folded into a cone-shaped form to match the wider diameter of the recipient left atrium. In a second step, cone’s superior margin was sutured to the superior vein’s atrial cuff, thereby creating a neoatrial cuff (Figs. 1B and 2). The bilateral lung transplantation was then completed in a usual fashion [5] and the patient was transferred to the intensive care unit, where he was extubated on the 1^st^ postoperative day. Following reconstruction, a high-dose prophylactic anticoagulation (anti-Factor Xa between 0.2 and 0.3 IU/ml) with unfractioned heparin was continued for 6 weeks. A ventilation/perfusion (V/Q) scan performed before discharge (day 29) showed a symmetrical perfusion of the donor lung (Fig. 3). Apart from an acute-on-chronic renal failure that improved upon conservative measurements and did not require renal replacement therapy, the patient made an uneventful recovery and was discharged home on the 30th postoperative day.

## Discussion and conclusions

An inadequate donor left atrial cuff is a rare technical issue after graft procurement for lung transplantation. Our case demonstrates that reconstruction of an inadequate cuff by biological patch repair is feasible in such cases. Various degrees of left atrial cuff insufficiency and reconstruction techniques have previously been described by Oto et al. in 2006 [2]. In our case, the lower pulmonary vein was circumferentially amputated. A cone-shaped neoatrial cuff was created by suturing a centrally cut out biological patch to the orifice, thereby successfully avoiding a right lower lobectomy. While biological grafts are commonly used in the field of cardiovascular surgery, their application in lung transplantation has rarely been reported [2]. As an alternative, donor pulmonary artery remnant or a donor pericardial patch can be used to create additional length and diameter of the venous cuff, [2]. In our case however, the defect was too large to be reconstructed by the remnant donor pulmonary artery. Nevertheless, it can be valuable to retain residual donor pericardium, pulmonary artery or parts of the superior vena cava during the back table preparation in case of a reconstruction. Further options to overcome the challenge of an insufficient cuff include the use of the donor pericardium surrounding the pulmonary venous confluence as a “pericardial skirt” [3, 6] or on the left side, a direct implantation into the left atrial appendage [7]. After reconstruction, signs of congestion should be closely monitored. Intraoperatively, the implanted lung should be assessed for darker discoloration or increasing consolidation. A constrained oxygenation or rising pulmonary arterial pressure can be suggestive for a venous obstruction and should be investigated by transesophageal echocardiography to distinguish reduced pulmonary venous flow from signs of cardiac failure or fluid overload [2]. Postoperatively, a contrast-enhanced CT or V/Q-scan can confirm an open anastomosis. Due to the transient renal insufficiency, a V/Q scan was preferred to verify the patency of the right inferior pulmonary vein in our case. Considering the high flow rate at the atrial anastomosis and since bovine pericardial repair does not require permanent anticoagulation, sub-therapeutic anticoagulation was administered for 6 weeks.

In order to avoid potential technical errors during graft procurement, a standardized protocol for donor lung procurement should be followed. A recently published consensus statement by the International Society for Heart and Lung Transplantation (ISHLT) addresses the standardization in the procurement process and particularly the surgical technique [4]. The consensus statement furthermore emphasizes the importance of an early evaluation of the organ and prompt communication of the findings to the transplant center [4].

Considering the shortage of suitable donor organs for lung transplantation, it is important to consider every donor organ for transplantation, even when facing technical difficulties. This case exemplifies a way of overcoming the challenge of an insufficient left atrial cuff by reconstruction with an acellular biological patch. The technique allows the formation of a neo-atrial cuff which can then be safely anastomosed to the recipient left atrium. Postoperative assessment by V/Q scan confirmed an unrestricted patency of the venous drainage in the affected lobe. On a second note, this case emphasizes the importance of careful inspection and evaluation of the donor lung immediately after procurement and early communication with the recipient implant team.

## Data Availability

Not applicable.

## Abbreviations

FEV1: Forced expiratory volume in 1 s
FVC: Forced vital capacity
CT: Computed tomography
V/Q scan: Ventilation/perfusion scan
